# Modulation of NK Cell Autocrine-Induced Eosinophil Chemotaxis by Interleukin-15 and Vitamin D_3_: A Possible NK-Eosinophil Crosstalk via IL-8 in the Pathophysiology of Allergic Rhinitis

**DOI:** 10.1155/2011/373589

**Published:** 2011-07-03

**Authors:** A. E. El-Shazly, P. P. Lefebvre

**Affiliations:** Division/GIGA Research, Department of Otolaryngology and Head and Neck Surgery-Rhinology, Liege University Hospital (Centre Hospitalier Universaitaire—CHU), Liege 4000, Belgium

## Abstract

Natural killer cells (NK) secrete eosinophilotactic cytokines, however, whether they contribute to eosinophil chemotaxis by secreting IL-8 is not known. We investigated the ability of CD56+CD3-ve (NK cells) to induce chemotaxis of peripheral blood eosinophils from allergic rhinitis (AR) patients, through IL-8 secretion, and the effects of IL-15, the NK cell proactivating cytokine, and calcitriol: 1*α*, 25-dihydroxy Vitamin D_3_ (vitamin D_3_), the immunomodulator agent, in this scenario. Herein, it is shown that supernatants from unstimulated NK cells exhibited chemotactic activity against eosinophil. This effect was significantly augmented by IL-15 (1 ng/mL) treatment, resulting in an increase in the chemotactic index of approximately 3 folds and was abrogated by neutralizing antibody (Ab) to IL-8 in a dose-dependent fashion. The amount of IL-8 secreted by NK cells was increased by IL-15 treatment from levels of 88.64 ± 21.5 to 178.9 ± 23.6 Pg/mL and was significantly reduced by 10^−6^ M vitamin D_3_ to levels of 59.2 ± 16.3 Pg/mL. Our results indicate a novel inflammatory crosstalk between NK cells and eosinophils via IL-15/IL-8 axis that can be modulated by vitamin D_3_.

## 1. Introduction

IL-8 is a potent activator for eosinophil chemotaxis in vitro and in vivo [1]. IL-8 also possesses a clear role in eosinophil chemotaxis in allergic respiratory diseases [2, 3]. On the other hand, IL-15 effect on human eosinophil is through inhibition of the spontaneous apoptosis via the autocrine production of GM-CSF and thus may perpetuate allergic inflammation by prolonged eosinophil survival [4]. Nonetheless, the role of IL-15 in the pathophysiology of allergic airways diseases is still in its infancy and remains a controversial issue [5–9].

NK cells activation plays a pivotal role in viruses and tumor lyses [10]; however, they also produce cytokines and thus are thought to play a proinflammatory role [11]. The secretagogue activity of NK cells includes IFN-*γ*, IL-10, TNF-*α*, MIP-1, MIP-1*β*, and GM-CSF. These cytokines are augmented by IL-15, a cytokine produced by activated monocytes/macrophages [12, 13]. Some investigators demonstrated the ability of NK cells to differentiate in the presence of IL-4 into NK cell subsets secreting distinct cytokines patterns similar to T_H2_ profile such as IL-5 and IL-13 [14, 15]. A more recent study demonstrated the existence of type 2 cytokine-secreting NK cells in AR and showed increased number and enhanced cytotoxicity of NK cells [16]. This highlights a novel role for NK cells in allergic diseases. Nonetheless, whether NK cells are able to attract eosinophils through IL-8 secretion is not known.

Increasing evidence supports a novel immunoregulatory role for vitamin D in allergic diseases such as asthma and AR [17, 18]. The ability of vitamin D to reduce cytokines from inflammatory cells is also well acknowledged. Therefore, the therapeutic potential of vitamin D as an anti-inflammatory agent in allergic diseases remains a subject of interest.

Herein, we show a novel crosstalk between human NK cell and eosinophil recruitment through IL-15/IL-8 axis. Results are discussed in relation to the possible novel therapeutic effect of vitamin D in downregulating this inflammatory event, and the possible role for NK cells in the pathophysiology of AR.

## 2. Materials and Methods

### 2.1. Cell Preparation

Eosinophils and NK cells were isolated by Percoll (66%) solution. NK cells were isolated from buffy coats to obtain large amount of cells for culture purposes, and eosinophils were freshly isolated from the peripheral blood of sixteen AR patients sensitized to different aeroallergens that were confirmed by skin tests and/or radioallergosorbent test (RAST). Eosinophils and NK cells were further purified by negative selection immunomagnetic cell separation (MACS; Miltenyi Biotec, Bergisch Gladbach, Germany), using anti-CD16 magnetic beads for eosinophils purification, and a cocktail of biotin-conjugated antibodies against lineage-specific antigens and a cocktail of microbeads (NK Cell Isolation Kit-human, Miltenyi Biotec, Bergisch Gladbach, Germany) for NK (CD56+ve CD3-ve) cells purification. Cells purity was >96% for both cell types. Cells viability was always >98% as judged by trypan blue.

### 2.2. Cell Culture

NK cells (1 × 10^6^ cells) were incubated in the presence or absence of 1 ng/mL IL-15 (R&D Systems; Minneapolis, MN), 10^−6^ M concentration of vitamin D_3_ (Cayman Chemical), 10^−5^ M concentration of H-89 Dihydrochloride (VWR-CALBIOCHEM), a PKA inhibitor, 10^−5^ M concentration of Bisindolylmaleimide (VWR-CALBIOCHEM), a PKC inhibitor, 10^−5^ M concentration of SB203580 (VWR-CALBIOCHEM), a P38 MAP-Kinase inhibitor, or 1 *μ*M concentration of Genistein (Sigma), a tyrosine kinase inhibitor, at 37°C-5% CO_2_ with 1 mL RPMI-1640 supplemented with 100 U penicillin/mL and 100 *μ*g streptomycin/mL (Lonza, Verviers, Belgium), 10% of inactivated fetal calf serum (Lonza) in 24 wells plate (BD biosciences). After 72 h incubation period, supernatants were carefully collected and analysed for their eosinophilotactic activity or IL-8 content. 

As for the measurement of IL-8 content induced by peripheral blood NK cells from AR patients before and after nasal challenge, peripheral blood NK cells were purified from additional four AR patients to house dust mite (HDM). After obtaining the patients consent, 30 mls of peripheral blood was obtained before and 6 hours after nasal challenge with HDM allergen. Purified NK cells at the concentration of 3 × 10^6^ cells/mL were incubated in the culture medium for 72 hrs. Supernatants were collected and the amount of IL-8 was measured by ELISA.

### 2.3. Chemotaxis Assays

Chemotaxis assays were performed in triplicate in a 48-well microchemotaxis Boyden chambers incubated in 5% CO_2_ at 37°C for 90 min. Aliquots of 29 *μ*L of the NK supernatant were placed in the lower wells and 50 *μ*L of eosinophils suspension (10^6^ cells/mL) were placed in the upper wells. The two chambers were separated by a 5.0 *μ*m pore polycarbonate membrane (Nuclepore, Whatman, Middlesex, UK). Migrated cells adherent to the lower surface were counted in 5 selected high power fields/well under a light microscope (5 hpf; X400). As for blocking experiments, NK supernatants were preincubated for 1 h and during the chemotaxis assay, with different doses (10^−8^–10^−6^ g/mL) of the monoclonal anti-IL-8Ab that neutralizes the biological activity of IL-8 (R&D systems; Minneapolis. MN).

### 2.4. Enzyme-Linked Immunosorbant Assay (ELISA)

The content of IL-8 was measured in supernatants of NK cells using human IL-8 cytoset Kit (Invitrogen corporation, USA) according to the manufacturers' recommendations.

### 2.5. Statistical Analysis

Statistical significance was performed by paired *t*-test. Values of *P* < 0.05 were considered significant.

## 3. Results

### 3.1. NK Cells Supernatants-Induced Eosinophil Chemotaxis

**Figure 1:**
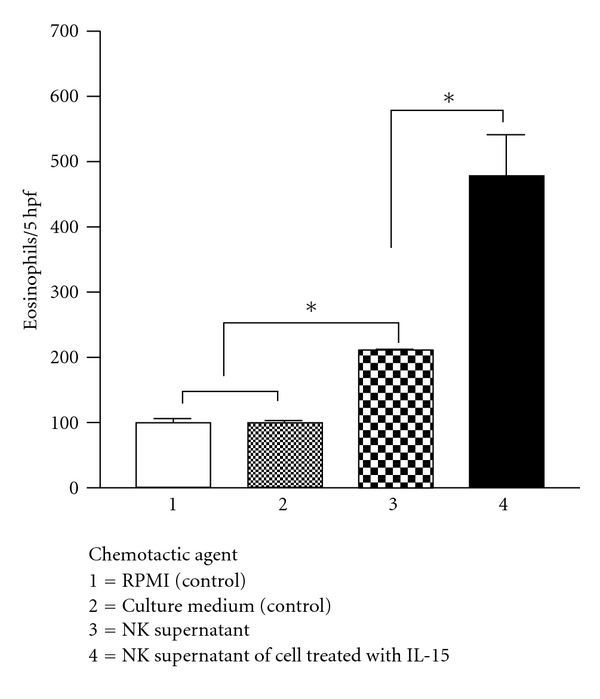
Eosinophilotactic activity of supernatants of resting NK cells versus supernatants of NK cells stimulated with IL-15. Results are the mean ± SEM of 10 independent experiments performed in triplicate. Asterisks indicate *P* < 0.05 by paired *t*-test.

NK cells supernatants induced chemotaxis of eosinophils from AR patients (Figure 1). NK cells treatment with the optimal dose of 1 ng/mL IL-15 during the culture period resulted in significant augmentation of NK cells supernatant-induced eosinophil chemotaxis from AR patients to almost 3-fold chemotaxis index. These results indicate a novel autocrine activity for NK cells in recruiting eosinophils and highlight a novel role for IL-15 in up-regulating this effect.

### 3.2. Blocking Activity of Anti-IL8 Ab

**Figure 2:**
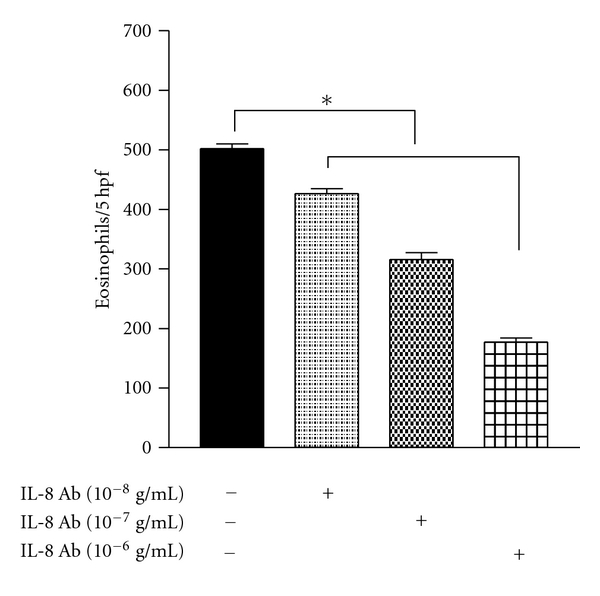
Effect of neutralizing IL-8 antibody on eosinophils chemotaxis against IL-15-induced NK cells supernatants. Results are the mean ± SEM of 6 independent experiments performed in triplicate. Asterisk indicates *P* < 0.05 by paired *t*-test.

Figure 2 shows that eosinophil chemotaxis of AR patients against NK supernatant of cells treated by IL-15 was significantly blocked by anti-IL-8 Ab. The blocking activity was in a dose-dependent fashion, but was not completely blocked to the control level. These results indicate that NK cells stimulated by IL-15 secrete eosinophilotactic chemokines that include at least in part IL-8 among others. To confirm this, we next measured by ELISA the amount of IL-8 spontaneously secreted by NK cells, and the modulatory effects of IL-15 and vitamin D_3_ on IL-8 secretion. 

### 3.3. Modulation of the Amount of Secreted IL-8 in NK Cells Supernatant by IL-15 and Vitamin D_3_

We tested the amount of IL-8 secreted by NK cells after 72 h culture period in the presence and absence of the optimal doses of IL-15 or vitamin D_3_. As can be seen in Table 1, cultured NK cells for 72 h secreted IL-8. The amount of recovered IL-8 was increased from 88.64 ± 21.5 to 178.9 ± 23.6 Pg/mL and was significantly reduced by vitamin D_3_ to 59.2 ± 16.3 Pg/mL. These results indicate the ability of IL-15 to upregulate the IL-8 secretagogue activity by NK cells, and the ability of vitamin D_3_ to significantly inhibit the IL-8 secretagogue activity of NK cells. Furthermore, it confirms the contribution of IL-8 to the eosinophilotactic cytokines secreted by NK cells. 

However, to gain insight into the possible signal pathway involved in NK cell induced-IL-8 modulation by IL-15 and vitamin D_3_, we treated NK cells with different kinases inhibitors during the culture period. H-89 Dihydrochloride, Bisindo-lylmaleimide, or Genistein did not modulate the amount of IL-8 secreted by NK cells (data not shown). However, as can be seen in Table 1, IL-8 secretion by NK cells was sensitive to SB203580 and resulted in reduction of IL-8 to 15 ± 0.5 Pg/mL. Similarly, the augmentation of IL-8 secretion by IL-15 was reduced by SB203580 to 54.75 ± 5.7. These results may indicate the involvement of P38 MAP-Kinase pathway in the signal transduction of IL-8 secretion by NK cells.

### 3.4. Modulation of the Amount of Secreted IL-8 in NK Cells Supernatant from AR Patients after Nasal Challenge

**Figure 3:**
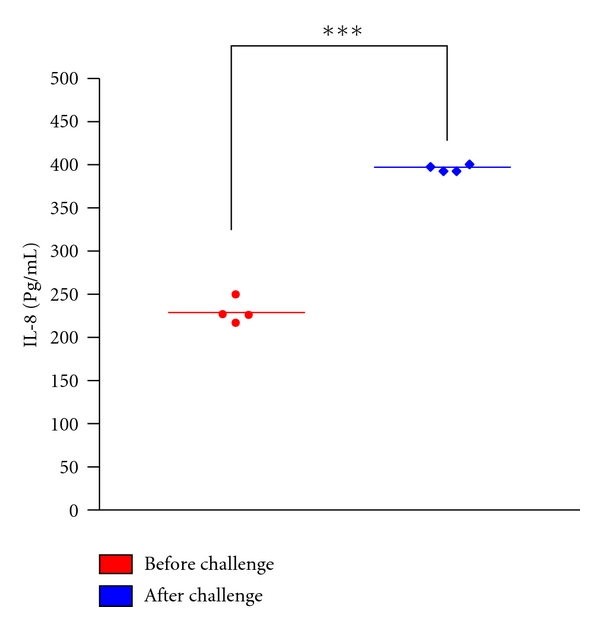
Amount of IL-8 secreted by NK cells of AR patients sensitized to HDM before and 6 h after nasal challenge with HDM allergen. Results are the mean ± SEM of 4 independent experiments. Asterisks indicate *P* < 0.05 by paired *t*-test.

Finally to further correlate our results to the pathophysiology of AR, we challenged AR patients to HDM with HDM allergen. NK cells purification before and at 6 h postchallenge were cultured for 72 h. Collected supernatants were checked by ELISA for the amount of IL-8 recovered. As demonstrated in Figure 3, after nasal challenge NK cells secreted a significantly higher amount of IL-8. This result indicates the importance of NK cells as a source for IL-8 in the pathophysiology of AR.

## 4. Discussion

In the current communication, we demonstrated a novel crosstalk between NK cells and eosinophils via IL-15/IL-8 axis. This may indicate a role for IL-15 in the pathophysiology of allergic rhinitis late-phase reaction and the promotion of eosinophilic inflammation. 

In AR disease, the mixed in vivo milieu of T_H2_ cytokines especially induced after allergen exposure prime eosinophils. This makes them exhibit distinct phenotype and exaggerated responses to chemokines such as IL-8 [19]. Herein, we show that nasal allergen challenge of AR patients also primes NK cells to secrete larger amounts of IL-8. Further, the ability of NK cells to secrete IL-8 in their supernatants that contributed to the attraction of eosinophils from AR patients may provide a further explanation to eosinophil recruitment in AR. 

IL-15 prolongs eosinophil survival and thus acts as a proeosinophilic inflammatory mediator [4]. In the current study, IL-15 demonstrated a further novel indirect role in helping eosinophil recruitment, at least in part through secretion of IL-8 from resting NK cells. 

Vitamin D binds to its nuclear receptor, the vitamin D receptor (VDR) through which it signals in its target cells. We demonstrated a significant inhibition of IL-8 by NK cells in the presence of Vitamin D_3_. This is consistent with an earlier study that demonstrated that vitamin D_3_ repressed IL-8 promoter activity induced by TNF-*α* in human melanoma cell line by 50% compared to 30% inhibition by dexamethasone, as well as TNF-*α*-induced IL-8 release and IL-8 mRNA level [20].

The fact that IL-8 secretion by NK cells was sensitive to P38 MAP-Kinase inhibition may indicate that IL-15 and vitamin D_3_ modulated IL-8 autocrine activity of NK cells through the same pathway. It is then reasonable to assume that IL-15 may induce IL-8 secretion by NK cells through stimulation of P38 MAP-kinase activity and that vitamin D_3_ reduces IL-8 secretion from NK cells through blocking effect on the P38 MAP-Kinase pathway. Nonetheless, further studies are needed to prove or disprove this hypothesis. In conclusion, our results may indicate the following:

1. a novel role for NK cells in the pathophysiology of AR through recruiting eosinophils,
2. a novel modulatory role for IL-15 in inducing eosinophils chemotaxis from AR patients through the NK cells secretagogue activity,
3. identifying IL-8 as an important cytokine that contributes to the eosinophilotactic agents secreted by NK cells,
4. indicating that vitamin D_3_ may be an effective therapeutic modality.

Our results open channels for other researchers to further elaborate on the exact mechanism(s) involved in NK-induced IL-8 modulation by IL-15 and vitamin D_3_ and P38 MAP-Kinase inhibition.

##  Conflict of Interests

The authors claim no conflict of interests with the current work.

## Figures and Tables

**Table 1:** Amount of IL-8 recovered in supernatants of NK cells after 72 h culture period.

Stimulant	IL-8 (Pg/mL) in NK cells supernatants
RPMI	88.6 ± 21.5
IL-15 (1 ng/mL)	178.9 ± 23.6
Vit D_3_ (10–6 M)	59.2 ± 16.3***
SB203580 (10–5 M)	15 ± 0.5***
IL-15 (1 ng/mL) + SB203580 (10–5 M)	54.75***

Results are ±SEM of 4–8 independent experiments. Asterisks indicate *P* < 0.05 by paired *t*-test.

## References

[1] Collins PD, Weg VB, Faccioli LH, Watson ML, Moqbel R, Williams TJ (1993). Eosinophil accumulation induced by human interleukin-8 in the guinea-pig in vivo. *Immunology*.

[2] Erger RA, Casale TB (1995). Interleukin-8 is a potent mediator of eosinophil chemotaxis through endothelium and epithelium. *American Journal of Physiology*.

[3] Lampinen M, Rak S, Venge P (1999). The role of interleukin-5, interleukin-8 and RANTES in the chemotactic attraction of eosinophils to the allergic lung. *Clinical and Experimental Allergy*.

[4] Hoontrakoon R, Chu HC, Gardai J (2002). Interleukin-15 inhibits spontaneous apoptosis in human eosinophils via autocrine production of granulocyte macrophage-colony stimulating factor and nuclear factor-*κ*B activation. *American Journal of Respiratory Cell and Molecular Biology*.

[5] Bierbaum S, Nickel R, Zitnik S (2006). Confirmation of association of IL-15 with pediatric asthma and comparison of different controls. *Allergy*.

[6] Aoi N, Masuda T, Murakami D (2006). IL-15 prevents allergic rhinitis through reactivation of antigen-specific CD8+ cells. *Journal of Allergy and Clinical Immunology*.

[7] Ruckert R, Brandt K, Braun A (2005). Blocking IL-15 prevents the induction of allergen-specific T cells and allergic inflammation in vivo. *The Journal of Immunology*.

[8] Ishimitsu R, Nishimura H, Yajima T, Watase T, Kawauchi H, Yoshikai Y (2001). Overexpression of IL-15 in vivo enhances Tc1 response, which inhibits allergic inflammation in murine model of asthma. *Journal of Immunology*.

[9] Kurz T, Strauch K, Dietrich H (2004). Multilocus haplotype analyses reveal association between 5 novel IL-15 polymorphisms and asthma. *Journal of Allergy and Clinical Immunology*.

[10] Sun PD (2003). Structure and function of natural-killer-cell receptors. *Immunologic Research*.

[11] Orange JS, Ballas ZK (2006). Natural killer cells in human health and disease. *Clinical Immunology*.

[12] Carson WE, Giri JG, Lindemann MJ (1994). Interleukin (IL) 15 is a novel cytokine that activates human natural killer cells via components of the IL-2 receptor. *Journal of Experimental Medicine*.

[13] Fehniger TA, Shah MH, Turner MJ (1999). Differential cytokine and chemokine gene expression by human NK cells following activation with IL-18 or IL-15 in combination with IL-2; implication for the innate immune response. *The Journal of Immunology*.

[14] Peritt D, Robertson S, Gri G, Showe L, Aste-Amezaga M, Trinchieri G (1998). Cutting edge: differentiation of human NK cells into NK1 and NK2 subsets. *Journal of Immunology*.

[15] Deniz G, Ajdis M, Aktas E, Blaser K, Akdis CA (2002). Human NK1 and NK2 subsets determined by purification of IFN-*γ*-secreting and IFN-*γ*-nonsecreting NK cells. *European Journal of Immunology*.

[16] Mesdaghi M, Vodjgani M, Salehi E (2010). Natural killer cells in allergic rhinitis patients and nonatopic controls. *International Archives of Allergy and Immunology*.

[17] Sandhu MS, Casale TB (2010). The role of vitamin D in asthma. *Annals of Allergy, Asthma and Immunology*.

[18] Erkkola M, Kaila M, Nwaru BI (2009). Maternal vitamin D intake during pregnancy is inversely associated with asthma and allergic rhinitis in 5-year-old children. *Clinical and Experimental Allergy*.

[19] Warringa RA, Mengelers HJ, Raiijmakers JA, Brijnzeel PL, Koenderman L (1993). Upregulation of formyl-peptide and interleukin-8-induced eosinophil chemotaxis in patient with allergic asthma. *Journal of Allergy and Clinical Immunology*.

[20] Harant H, Andrew PJ, Reddy GS, Foglar E, Lindley IJD (1997). 1*α*,25-dihydroxyvitamin D_3_ and a variety of its natural metabolites transcriptionally repress nuclear-factor-*κ*B-mediated interleukin-8 gene expression. *European Journal of Biochemistry*.

